# Uterine Leiomyomas and Infertility: A Comparison of National and International Guidelines

**DOI:** 10.7759/cureus.50992

**Published:** 2023-12-23

**Authors:** Georgios Michos, Themistoklis Dagklis, Evangelos Papanikolaou, Ioannis Tsakiridis, Kyriakos Oikonomou, Apostolos M Mamopoulos, Ioannis A Kalogiannidis, Apostolos Athanasiadis

**Affiliations:** 1 3rd Department of Obstetrics and Gynecology, Aristotle University of Thessaloniki, Thessaloniki, GRC

**Keywords:** infertility, guidelines, myomectomy, fibroids, leiomyomas

## Abstract

Uterine leiomyomas are the most common benign tumors of the female genital track, causing various symptoms and problems, including a possible impact on fertility. The relationship between fibroids and infertility has long been a debate among gynecologists. Management of fibroids in women with otherwise unexplained infertility worldwide lacks standardized, evidence-based guidelines. Therefore, a review of guidelines from the Royal Australian and New Zealand College of Obstetricians and Gynecologists, the American College of Obstetricians and Gynecologists, the Society of Obstetricians and Gynecologists of Canada, the Collège National des Gynécologues et Obstétriciens Français, and the American Society of Reproductive Medicine was conducted. There is agreement among all guidelines that the effect of fibroids on fertility is related to their position in the uterus and the alteration of the endometrial cavity. However, whether surgical intervention (laparotomy, laparoscopy, or hysteroscopy) is required varies among committees. More specifically, for submucous myomas, all guidelines agree that surgical intervention is needed. On the other hand, regarding intramural myomas, there is no consensus on what the approach may be. Novel treatments such as uterine artery embolization (UAE) and magnetic resonance-guided focused ultrasound surgery (MRgFUS) should only be used in clinical trial settings. Nevertheless, all guidelines agree that medical management of fibroids further delays efforts to conceive and has no role as a stand-alone treatment of fibroids; though, the use of GnRH analogues preoperatively can be useful to improve anemia and/or reduce fibroid volume. There is a need for updated international protocols to be introduced, in order to help clinicians dealing with fibroids and infertility to better suggest the optimal treatment.

## Introduction and background

Uterine fibroids (also known as leiomyomas) are the most prevalent pelvic tumors in women [1,2]. They originate from the smooth muscle cells and the fibroblasts of the myometrium and can be classified as submucosal, intramural, or subserosal, depending on their location. They most commonly arise in women of reproductive age, and they can either present as symptomatic (e.g., abnormal uterine bleeding, pelvic pain, or pressure) and/or have a negative impact on reproductive and pregnancy outcomes [3,4].

There is no classification widely accepted to categorize fibroids and leiomyomas are usually classified according to their relationship to the endometrium and myometrium. Additional factors such as size, number, and whether are symptomatic or not could have a prognostic role as well [5].

In 2011, the International Federation of Gynecology and Obstetrics (FIGO) proposed the allocation of leiomyomas into eight subtypes, from 0 to 8, aiming for a more detailed classification [6]. According to this classification, submucosal myomas are subdivided into three categories (0, 1, 2) depending on the percentage of the fibroid protruding from the endometrial cavity. Intramural myomas can be subdivided into three categories (3, 4, 5) and subserosal into 6, 7, 8 (Table 1).

**Table 1:** Leiomyoma classification system FIGO: International Federation of Obstetrics and Gynecology

Classification	Characteristics	FIGO Subtype
Submucosal	100% intracavitary	0
	<50%	1
	>50%	2
Intramural	Contacts endometrial cavity	3
	Intramural	4
	Mainly intramural (<50% subserosal)	5
Subserosal	Mainly subserosal (<50% intramural)	6
	Pedunculated	7
	Other (cervical, parasitic)	8

Myomas are common findings in women of reproductive age. In addition, it is well-known that infertility can be caused by many different factors and sometimes more than one factor coexist. The combination of high fibroid prevalence and possible contribution to reproductive outcomes such as infertility and recurring pregnancy loss demonstrates the paramount importance of international protocols regarding the management of women with leiomyomas and otherwise unexplained infertility. However, the available data evaluating reproductive outcomes, come from observational studies and, thus, are predisposed to selection bias and confounders such as increased prevalence with increased age. 

The aim of this review was to summarize and compare recommendations from five national and international societies on fibroids and infertility, regarding their medical and surgical management.

## Review

The most recently published guidelines on fibroids and their possible impact on fertility were retrieved and a descriptive review was conducted. Five national guidelines were identified from the Royal Australian and New Zealand College of Obstetricians and Gynecologists (RANZCOG), the American College of Obstetricians and Gynecologists (ACOG), the Society of Obstetricians and Gynecologists of Canada (SOCG), the Collège National des Gynécologues et Obstétriciens Français (CNGOF), and the American Society of Reproductive Medicine (ASRM) [7-11]. An overview of the recommendations is presented in Table 2.

**Table 2:** Summary of the recommendations on fibroids in infertile women RANZCOG: Royal Australian and New Zealand College of Obstetricians and Gynecologists; ACOG: American College of Obstetricians and Gynecologists; SOCG: Society of Obstetricians and Gynecologists of Canada; CNGOF: French College of Gynecologists and Obstetricians; ASRM: American Society for Reproductive Medicine; MRI: Magnetic Resonance Imaging; TVS: Transvaginal Ultrasound

	RANZCOG	ACOG	SOCG	CNGOF	ASRM
Country	Australia and New Zealand	United States	Canada	France	United States
Issued	March 2018	August 2008	March 2015	July 2012	June 2017
Title	Fibroids in Infertility	Alternatives to Hysterectomy in the Management of Leiomyomas	The Management of Uterine Fibroids in Women with Otherwise Unexplained Infertility	Therapeutic Management of Uterine Fibroid Tumors: Updated French Guidelines	Removal of Myomas in Asymptomatic Patients to Improve Fertility and/or Reduce Miscarriage Rate: a Guideline
Pages	7	14	9	10	10
References	2	117	52	61	59
Evaluation of uterine fibroids	Optimal imaging techniques MRI, sonohysterography or hysteroscopy, hysterosalpingography, and TVS are insufficiently sensitive or specific	Not discussed	MRI is the most reliable method. TVS, hysterosalpingography, hysterosonography, hysteroscopy are alternatives.	Not discussed	Not discussed

Evaluation of uterine fibroids

Since the connection between fibroids and infertility lies mainly in their position and relation to the uterine cavity, efforts should be made to adequately assess the uterine cavity and its relations with the existing fibroids. Both the RANZCOG and the SOCG agree that MRI is the most reliable technique available. Additional evaluation tools such as transvaginal ultrasound (TVS), hysterosonography, hysterosalpingography, and hysteroscopy offer valuable information and should be considered on each patient separately; however, the RANZCOG comments that hysterosalpingography and TVS lack sensitivity or specificity, whereas hysteroscopy may under-evaluate certain submucosal myomas, due to the increased intrauterine pressure during the procedure, altering the outline of the uterus (Table 2) [7].

Effect of fibroid position on fertility outcomes

There is a universal consensus among all guidelines that the relative position of a fibroid on the uterus may affect fertility and pregnancy outcomes. Uterine leiomyomas can transform the normal anatomy of the uterus, thus possibly affecting implantation and gestation. According to all the guidelines, the presence of subserosal myomas has not been linked to a negative impact on a couple’s ability to conceive. Additionally, submucosal fibroids are associated with reduced fertility and pregnancy complications, since this type of fibroids is most likely to invade the uterine cavity (Table 3). Regarding intramural fibroids, only the CNGOF correlates them with a negative impact on spontaneous and assisted pregnancy rates [10]. The remaining four guidelines state that the association between intramural fibroids and fertility lies in the degree of endometrium involvement and the disruption of its contour. The SOCG remarks that upon no endometrium impingement, the effect on fertility is minimal [9].

**Table 3:** Effect of fibroid position on fertility outcome RANZCOG: Royal Australian and New Zealand College of Obstetricians and Gynecologists; ACOG: American College of Obstetricians and Gynecologists; SOCG: Society of Obstetricians and Gynecologists of Canada; CNGOF: French College of Gynecologists and Obstetricians; ASRM: American Society for Reproductive Medicine; SS: Subserosal; IM: Intramural; SM: Submucosal

	RANZCOG	ACOG	SOCG	CNGOF	ASRM
Effect of fibroid position on fertility outcomes	SS fibroids do not appear to have significant effect on fertility outcomes. IM fibroids may be associated with reduced fertility outcomes. SM fibroids are associated with reduced fertility and increased miscarriage rate	SM and IM fibroids can cause distortion or obstruction of the uterine cavity or cervical canal and, thus, may affect fertility or lead to pregnancy complications. SS fibroids have not been shown to have an impact on reproductive outcome	SS fibroids do not appear to have an impact on fertility. IM fibroids have a small impact and are even less significant when the endometrium is not involved. SM fibroids have a negative impact on rates of implantation, clinical pregnancy, miscarriage	SM and IM fibroids have a negative impact on both spontaneous and assisted pregnancy rates. SS fibroids do not appear to have an impact on fertility	Insufficient evidence to determine that a specific myoma, size, number, or location (excluding submucosal myomas or intramural myomas impacting the endometrial cavity contour) is associated with a reduced likelihood of achieving pregnancy or an increased risk of early pregnancy loss

Indications for myomectomy in infertile women

Comparing the guidelines, there is no consensus regarding the indications for surgical intervention in women with fibroids and otherwise unexplained infertility (Table 4). The RANZCOG and the ACOG link the decision for intervention to the success of Assisted Reproductive Technology (ART) and improving such techniques. Thus, according to RANZCOG, women undergoing ART with submucosal fibroids, and couples presenting with multiple failed cycles of ART where the female partner has intramural fibroids, constitute an indication for myomectomy [7]. Furthermore, fibroids distorting the uterine cavity should be excised before infertility treatment, and in women with several unsuccessful in vitro fertilization (IVF) cycles, despite adequate ovarian response and good quality embryos, the presence of leiomyomas justifies surgical intervention, as per ACOG [8]. 

According to the CNGOF and the RANZCOG, the presence of symptoms related to fibroids can lead clinicians to offer surgical management. Although data is insufficient as to whether myomectomy offers a clear fertility benefit, the prevalence of symptoms, such as vaginal bleeding or pressure symptoms, accounts for the intervention as per RANZCOG. The CNGOF states that intramural fibroids should be surgically removed if they are symptomatic [10].

Apart from the RANZCOG, the rest of the guidelines point out that fibroid position and size, potentially altering the contour of the endometrium and adversely affecting fertility, pose an indication for surgical intervention. In particular, the ASRM suggests that myomectomy may be considered in such circumstances, i.e., severe distortion of the pelvic architecture, complicating access to the ovaries for oocyte retrieval, or asymptomatic women with submucosal or intramural fibroids, impinging the endometrial cavity [11-13]. Regarding size alone, submucosal fibroids less than 4 cm should be removed regardless of the presence of symptoms, according to CNGOF, or if a rapid growth of fibroid size has been documented, according to ACOG. Finally, the SOCG recommends that infertile patients with large (>5 cm) Type II submucosal fibroids or Type II fibroids with <1 cm between the external surface of the fibroid and the uterine serosa ought to be managed by abdominal myomectomy (laparoscopic or laparotomy), while cut off point for hysteroscopic myomectomy is 5 cm (depending on surgeons’ expertise) [14,15].

**Table 4:** Indications for myomectomy in Infertile women RANZCOG: Royal Australian and New Zealand College of Obstetricians and Gynecologists; ACOG: American College of Obstetricians and Gynecologists; SOCG: Society of Obstetricians and Gynecologists of Canada; CNGOF: French College of Gynecologists and Obstetricians; ASRM: American Society for Reproductive Medicine; ART: Assisted Reproductive Technology; IVF: In Vitro Fertilization; IM: Intramural; SM: Submucosal

	RANZCOG	ACOG	SOCG	CNGOF	ASRM
Indications for myomectomy in infertile women	Women undergoing ART who have demonstrated SM fibroid(s). Infertile women with symptomatic fibroid(s). Couples presenting with multiple failed cycles of ART where the female partner has IM fibroids.	Distorted uterine cavity caused by leiomyomas. Women with several unsuccessful IVF cycles despite appropriate ovarian response and good-quality embryos. Rapidly growing leiomyomas	Infertile women with large (>5 cm) Type II submucosal fibroids or Type II fibroids with <1 cm between the external surface of the fibroid and uterine serosa	SM fibroids are less than 4 cm regardless they are symptomatic. IM fibroids myomectomy only for symptomatic	In asymptomatic women with cavity-distorting myomas myomectomy may be considered to improve pregnancy rates; Special circumstances, i.e., severe distortion of the pelvic architecture complicating access to the ovaries for oocyte retrieval

The role of medical management

Apart from the ASRM, the guidelines allude to the medical management of fibroids. There is a consensus that there is no role for medical management as a stand-alone treatment in the infertile population since it is associated with suppression of ovulation, reduction of estrogen levels, and, thus, an additional hindrance in couples’ ability to conceive. The use of GnRH analogs preoperatively should be evaluated in each patient individually in terms of cost and potential side effects, i.e., reduced bone density. Nevertheless, the use of such medication prior to surgery can be useful to improve hematological parameters and reduce tumor volume. Finally, the RANZCOG addresses that ulipristal acetate should not be used due to significant adverse effects (liver failure-regular liver monitoring recommended) (Table 5) [16,17].

**Table 5:** The role of medical management RANZCOG: Royal Australian and New Zealand College of Obstetricians and Gynecologists; ACOG: American College of Obstetricians and Gynecologists; SOCG: Society of Obstetricians and Gynecologists of Canada; CNGOF: French College of Gynecologists and Obstetricians; ASRM: American Society for Reproductive Medicine; GnRH: Gonadotropin-releasing Hormone

	RANZCOG	ACOG	SOCG	CNGOF	ASRM
The role of medical management	Not recommended for the management of infertility associated with fibroids. Short-term use of a GnRH analog can be useful for pre-operative correction of anemia or short-term reduction in fibroid volume. The use of ulipristal acetate is not recommended due to the risk of significant adverse side effects	Benefits of preoperative use of GnRH agonists should be weighed against their cost and side effects	No role for medical therapy as a stand-alone treatment	GnRH analogs can be used for preoperative management	Not discussed

Surgical approach

As mentioned in the RANZCOG, ACOG, SOCG, and CNGOF guidelines, the surgical approach varies between laparotomy, laparoscopy, and hysteroscopy depending on the location and the size of the fibroid (Table 6). In particular, the hysteroscopic method is recommended for the management of submucosal leiomyomas as per ACOG and ASRM, with a restriction of maximum size of 5 cm. Even though larger fibroids have also been treated hysteroscopically, it has the risk of either incomplete tumor removal or procedure repetition in the future according to SOCG [18,19].

**Table 6:** Surgical management RANZCOG: Royal Australian and New Zealand College of Obstetricians and Gynecologists; ACOG: American College of Obstetricians and Gynecologists; SOCG: Society of Obstetricians and Gynecologists of Canada; CNGOF: French College of Gynecologists and Obstetricians; ASRM: American Society for Reproductive Medicine; GnRH: Gonadotropin-releasing Hormone

	RANZCOG	ACOG	SOCG	CNGOF	ASRM
Surgical approach	Laparotomy, laparoscopy, or hysteroscopy, depending on the size and location of the fibroid.	Laparotomy, laparoscopy, or hysteroscopy, depending on the size and location of the fibroid; Hysteroscopic management for SM leiomyomas.	Laparotomy, laparoscopy, or hysteroscopy depending on the size and location of the fibroid; SM fibroids should be managed hysteroscopically (size <5cm, although larger have been managed, often repeat procedures are necessary)	Laparotomy, laparoscopy, or hysteroscopy depending on the size and location of the fibroid.	Fair evidence that hysteroscopic myomectomy for submucosal fibroids improves clinical pregnancy rates; Insufficient evidence to conclude that hysteroscopic myomectomy reduces the likelihood of early pregnancy loss in women with infertility and a submucous fibroid

Other treatments

All the guidelines mention less invasive, and uterus-sparing techniques as alternatives to myomectomy in the management of leiomyomas. Apart from the ACOG that recommends uterine artery embolization (UAE) and magnetic resonance-guided focused ultrasound surgery (MRgFUS) as a safe alternative to hysterectomy, the other guidelines are hesitant to recommend such techniques due to insufficient data. Thus, UAE, MRgFUS, radio-frequency ablation (RFA), myolysis, and permanent or temporary uterine artery ligation should not be used outside the setting of clinical trials according to RANZCOG, CNGOF, and ASRM. In fact, the SOCG guideline goes as far as to highlight that UAE is contraindicated in women desiring future pregnancy since this approach has been associated with a reduction of ovarian reserve and worse pregnancy and miscarriage rates (Table 7) [20,21].

**Table 7:** Other treatments RANZCOG: Royal Australian and New Zealand College of Obstetricians and Gynecologists; ACOG: American College of Obstetricians and Gynecologists; SOCG: Society of Obstetricians and Gynecologists of Canada; CNGOF: French College of Gynecologists and Obstetricians; ASRM: American Society for Reproductive Medicine; UAE: Uterine Artery Embolization; MRgFUS: Magnetic Resonance Guided Focused Ultrasound Surgery; RFA: Radio Frequency Ablation

	RANZCOG	ACOG	SOCG	CNGOF	ASRM
Other treatments	UAE, MRgFUS, myolysis, permanent or temporary uterine artery ligation, and RFA should only be used in the setting of approved clinical trials on the management of fibroids in women with infertility	UAE and MRgFUS as alternative to hysterectomy in the management of leiomyomas	UAE contraindicated	UAE not recommended MRgFUS: only in the setting of clinical trials	UAE not recommended

Postoperative adhesions

The SOCG and the CNGOF emphasize the contribution of adhesions postoperatively in infertility. Therefore, they suggest measures to prevent synechiae formation. Specifically, the SOCG guideline recommends that, upon abdominal resection, the surgeon implements an anterior uterine incision, if possible, to minimize the formation of postoperative synechiae. Finally, according to the CNGOF, for the surgical treatment of submucosal fibroids, the use of bipolar energy and antiadhesion gel reduces the risk of adhesion formation and improves pregnancy rates (Table 8).

**Table 8:** Postoperative adhesions RANZCOG: Royal Australian and New Zealand College of Obstetricians and Gynecologists; ACOG: American College of Obstetricians and Gynecologists; SOCG: Society of Obstetricians and Gynecologists of Canada; CNGOF: French College of Gynecologists and Obstetricians; ASRM: American Society for Reproductive Medicine

	RANZCOG	ACOG	SOCG	CNGOF	ASRM
Adhesions	Not discussed	Not discussed	Use of an anterior uterine incision (if possible) to minimize the formation of postoperative adhesions.	The use of bipolar energy and antiadhesion gel to reduce the risk of postoperative synechiae and improve pregnancy rates	Not discussed

## Conclusions

This review has shown that there is an agreement between the reviewed guidelines on the effect of fibroids and fertility; (i) submucosal fibroids do impair reproductive outcomes, especially ones significantly altering the normal outline of the uterus, (ii) when dealing with intramural fibroids, the RANZCOG, the SOCG, the ACOG, and the ASRM correlate impingement of the uterine cavity and negative effect on fertility, (iii) subserosal fibroids do not appear to impact fertility. The presence of myomas, regardless of location, significantly decreases both implantation and clinical pregnancy rates. Furthermore, the main issue of debate is when the decision for surgical management of infertile women with fibroids should be taken to increase their probability of conceiving. Even though data differs among the guidelines, surgical treatment should aim for an immaculate uterine cavity, improving thus both spontaneous and IVF pregnancy rates. It is recommended that the technique implemented in each patient should be decided by the surgeon based on the size and the location of the fibroid. On the other hand, the impact of the number and size of leiomyomas on fertility has not been clearly described; however, reproductive outcomes seem to be related to fibroid location. The development of newer, less-invasive, approaches (UAE, MRgFUS, RFA, myolysis, temporary or permanent uterine artery ligation) has not yet been thoroughly evaluated and none of the guidelines (except ACOG) recommends such techniques outside of the settings of clinical trials. In conclusion, the development of evidence-based protocols requires more studies, taking into consideration the possible selection bias that affects results and leads to inconclusive data. The high prevalence of leiomyomas and their possible negative influence on fertility underscores the necessity for such new protocols or, if possible, a guideline algorithm to guide clinicians, effectively optimize the management of such patients, and improve their reproductive outcomes.
